# Association and comparison of periodontal and oral hygiene status with serum HbA1c levels: a cross-sectional study

**DOI:** 10.1186/s12903-023-03042-7

**Published:** 2023-07-02

**Authors:** Abid Rahim, Sabreen Hassan, Naeem Ullah, Nawal Noor, Ahmed, Rimsha Rafique, Farhad Ali Khattak, Saima Afaq

**Affiliations:** 1 Sardar Begum Dental College, Gandhara University, Peshawar, Pakistan; 2 Dental Surgery Department, Qazi Hussain Ahmed Medical Complex (MTI), Nowshera, Pakistan; 3 Department of Oral Pathology, Saidu College of Dentistry, Swat, Pakistan; 4 Department of Community Medicine, Saidu Medical College, Swat, Pakistan; 5 Superior University, Lahore, Pakistan; 6 Research & Development Cell, Khyber College of Dentistry, Peshawar, Pakistan; 7 Institute of Public Health and Social Sciences, Khyber Medical University, Peshawar, Pakistan; 8 School of Public Health Faculty of Medicine, Imperial College London, London, UK

**Keywords:** Periodontitis, Diabetes mellitus, Probe, Bleeding on probing, Pocket depth, Loss of attachment

## Abstract

**Background:**

Diabetes Mellitus and periodontitis are chronic diseases with known reciprocal association. Studies have shown that uncontrolled diabetes increases the risk of development and progression of periodontal disease. This study aimed to explore the association and severity of periodontal clinical parameters and oral hygiene with HbA1c levels in non-diabetics and T2DM patients.

**Materials and methods:**

In this cross-sectional study, the periodontal status of 144 participants, categorized into non-diabetics, controlled T2DM, and uncontrolled T2DM and were assessed via the Community Periodontal Index (CPI), Loss of Attachment Index (LOA index), and the number of missing teeth, while oral hygiene was measured by utilizing the Oral Hygiene Index Simplified (OHI-S). SPSS was used for data analysis. Chi-square test was used to find out the association of different independent variables with HbA1c groups, while ANOVA and post-hoc tests were run for inter-group and intra-group comparison respectively.

**Results:**

Out of 144 participants, the missing dentition was prevalent in uncontrolled T2DM with mean 2.64 ± 1.97 (95% CI 2.07–3.21; p = 0.01) followed by controlled T2DM 1.70 ± 1.79 (95% CI 1.18–2.23; p = 0.01) and non-diabetics 1.35 ± 1.63 (95% CI 0.88–1.82; p = 0.01) respectively. Furthermore, non-diabetics had a higher proportion of CPI score 0 (Healthy) [30 (20.8%); p = 0.001] as compared to uncontrolled T2DM [6 (4.2%); p = 0.001], while CPI score 3 was more prevalent in uncontrolled T2DM in comparison to non-diabetics. Loss of attachment (codes-2,3 and 4) was also frequently observed in uncontrolled T2DM compared to non-diabetics (p = 0.001). Similarly, based on Oral Hygiene Index- Simplified (OHI-S), the result showed that poor oral hygiene was most commonly observed in uncontrolled T2DM 29 (20.1%) followed by controlled T2DM patients 22 (15.3%) and non-diabetic [14 (9.7%); p = 0.03].

**Conclusion:**

This study showed that periodontal status and oral hygiene status were deteriorated in uncontrolled T2DM patients compared to non-diabetic participants and controlled T2DM.

**Supplementary Information:**

The online version contains supplementary material available at 10.1186/s12903-023-03042-7.

## Introduction

Diseases of the periodontium (i.e., gingivitis and periodontitis) are multifactorial bacterial diseases of the soft and hard tissues encompassing and supporting the teeth. It is initiated via the aggregation of a pathogenic dental plaque on the tooth surface, and inside which microbial dysbiosis prompts a constant non-resolving damaging provocative reaction [1]. The global prevalence of periodontitis ranges from 15 − 47% and in its most severe form affects 10.8% of the population [2]. It has been ranked seventh and 32nd worldwide for prevalence and incidence respectively [3]. Chronic periodontal disease not only affects the oral health but also systemic health [4]. Studies by Isola et al. found that periodontitis patients showed a higher risk of developing endothelial dysfunction and cardiovascular disease [5, 6]. The development and progression of periodontal diseases are directly linked to oral hygiene, and maintaining good oral hygiene reduces the risk of periodontitis [7, 8]. Fair and poor oral hygiene maximize the risk of periodontitis by two to five times respectively [9].

Diabetes mellitus (DM) portrayed by hyperglycemia, is a metabolic disorder caused by insufficient insulin production, insufficient insulin activity, or both. Diabetes Type-1 is characterized by an autoimmune attack on the pancreatic insulin-producing β cells, which leads to insufficient insulin synthesis. Type-2 diabetes mellitus is brought about by a blend of insulin opposition and insulin emission disability. Diabetes is currently thought to affect over 10% of people worldwide. According to estimates, 462 million people worldwide or 6.28% of the world’s population have T2DM [10, 11].

The relationship between diabetes and periodontitis is symbiotic because persistent hyperglycemia has been shown to negatively influence oral health, and severe periodontitis can have a negative impact on both glycemic control and diabetic complications [12]. Advanced glycation end-products are produced by type 2 diabetes and impaired insulin sensitivity, which leads to the generation of inflammatory cytokines and predisposes individuals to inflammatory conditions like periodontitis [13]. Diabetic individuals with periodontitis exhibited higher amounts of inflammatory mediators in their saliva and their crevicular fluid than non-diabetics with periodontitis, including numerous kinds of cytokines [14]. There is a proportionate association between HbA1c and periodontitis [15, 16]. On the other hand, periodontitis adds to the overall inflammatory load in the body by the transfer of bacteria and their products, cytokines, and inflammatory mediators via breached pocket epithelium and higher GCF miRNAs expression, exacerbating the development of complications in diabetic patients [5, 14, 17].

Given the well-established link between periodontitis and type 2 diabetes mellitus, this cross-sectional study aims to evaluate and compare the periodontal and oral hygiene status of individuals having different levels/categories of HbA1c. Unlike previous studies, this research simultaneously investigates periodontitis, oral hygiene, and diabetes in three subgroups: non-diabetics, controlled, and uncontrolled type 2 diabetes patients. By investigating this triad, this study provides a comprehensive understanding of the relationship between oral health and diabetes, which has a significant impact on overall well-being. Moreover, it aims to provide valuable insights into the extent of periodontal disease and oral hygiene status in a population specific to low and middle-income countries. The results of this study may have important implications for the prevention and management of periodontitis and type 2 diabetes, particularly in resource-limited settings.

## Materials and methods

### Study participants

This cross-sectional study was conducted on patients visiting the outpatient department of Qazi Hussain Ahmed Medical Complex (Medical Teaching Institute/MTI), Khyber Pakhtunkhwa - Pakistan from March 2021 to June 2021, after ethical approval from Khyber Medical University (DIR/KMU-EB/EC/00080/DR). Using the WHO sample size calculator, considering the frequency of poor oral hygiene status among diabetics P1(22% compared to poor oral hygiene among non-diabetics P2 (37%), taking 80% power of the test and 5% margin of error, a total of 144 patients were recruited [18]. Based on the HbA1c levels, all of the participants were divided into three equal groups. *Group A*: Non-diabetic (HbA1c ≤ 5.7%) *Group B*: Controlled T2DM (HbA1c 6.0–6.9%) *roup C*: Uncontrolled T2DM (HbA1c ≥ 7%) [19]. The enrolment criteria were (i) Dentate patients with age 20–60 years. (ii) Subjects diagnosed with T2DM for more than one year (groups B and C). The exclusion criteria were (i) Patients having any other systemic disease/s other than diabetes mellitus. (ii) individuals who have undergone periodontal therapy in the past three months (iii) Patients who have taken antibiotics for the past month. (iv) Patients having 3rd molar impaction, endodontic problems, or limited mouth opening. (v) Female participants that are expecting/lactating.

### Interview questionnaire

Each participant gave written informed consent, after being informed about the pertinent interview and oral examination required for carrying out the study. An organized form was used to obtain the necessary information like age, gender, education level, occupation, frequency of tooth cleaning and tool used for cleaning, last dental visit, the reason for a dental visit, frequency and type of tobacco consumed, duration of diabetes, and complications of diabetes, number of missing teeth. The HbA1c level for each T2DM patient was obtained from his/her medical record. Similarly, the HbA1c level for non-diabetics was determined before the oral examination.

### Oral examination

All the subjects underwent oral examination to evaluate their periodontal status (i.e., bleeding upon probing, depth of the pocket, and loss of attachment), and oral hygiene status (dental plaque and calculus). The examination for each participant was done via mouth mirror, CPITN-C probe, and No. 5 explorer (Shepherd’s Crook). The periodontal assessment was done using the Community Periodontal Index (CPI index) (with scoring criteria as code-0, code-1, code-2, code-3, code-4, or code-x) and the Loss of Attachment Index. Oral hygiene for each patient was assessed according to Oral Hygiene Index-Simplified and categorized into Good oral hygiene, Fair, and Poor oral hygiene. Indexed teeth: 17/16, 11, 26/27 (buccal surfaces) and 47/46, 31, 36/37 (lingual surfaces) were examined during the oral examination. Oral examination was carried out by a single and trained periodontist (AR) to prevent differences in measurements.

### Data analysis

All the relevant data were collected and recorded on a preformed structured questionnaire. Statistical analysis was done via SPSS version 25. The Chi-square test/Fsher exact test was used to compare non-diabetics, controlled T2DM, and uncontrolled T2DM with other explanatory variables like sociodemographic traits, diabetes-associated factors, oral hygiene status, and periodontal status, while one-way ANOVA was carried out to determine any statistical significance among the three groups in relation to other continuous variables. For those with significant P value, Post Hoc (Tukey HSD) Test was run to explore the mean differences between the pair of groups. The threshold for statistical significance was set at p ≤ 0.05.

## Results

### Baseline characteristics

A total of 144 participants voluntarily took part in this study with 87 (59.7%) males, with a mean age of 42.5 ± 12.3 years for non-diabetic and 43.7 ± 10.2 years for controlled diabetics while 49.2 ± 8.8 years for uncontrolled T2DM (p = 0.002) and mean serum HbA1c levels of 5.2 ± 1.09, 6.7 ± 0.50 and 8.3 ± 1.09 for the three groups (non-diabetics, controlled T2DM, uncontrolled T2DM) respectively (p = 0.001) (Tables 1 and 2). The prevalence of missing dentition was comparatively higher in the uncontrolled T2DM group with mean of 2.64 ± 1.9 and 1.3 ± 1.6 (Table 2). The mean difference of various variables was notably different between non-diabetics and uncontrolled T2DM (Table 3). The majority of the participants were illiterate 44 (30.6%), while only 32 (22.2%) had completed their graduation. The majority of the participants 47 (32.6%) were nonoccupational, followed by employed participants 41(28.55). Most of the participants 48 (33.3%) cleaned their teeth once a day, twice daily 32 (22.2%), and once a week by 33 (22.9%) participants. Toothbrush was used as a cleaning tool by 101 (70.1%), miswak was used by 22 (15.3%), and 21 (14.6%) participants used other tools (*danddassa*/Juglans regia linn) for cleaning their teeth. A minor number of participants 13 (9.0%) also had no dental check-up throughout their life. Most of the participants 91 (63.2%) did not use any tobacco product, and 22 (15.3%) consumed smoked tobacco (cigarette), while 31 (21.5%) used smokeless tobacco (*naswar*). Among the tobacco consumers, 44 (30.6%) consumed tobacco ≥ 3 times a day (Table 1).

**Table 1:** Association of Diabetic and Non-diabetic group(s) with different explanatory variables

Characteristics	Categories	Groups			P-value
		Non-diabetics (n = 48)	Controlled T2DM (n = 48)	Uncontrolled T2DM (n = 48)	
**Gender**	Male	26 (18.1%)	32 (22.2%)	28 (19.4%)	0.44
	Female	22 (15.3%)	16 (11.1%)	20 (13.9%)	
**Education level**	Illiterate	11 (7.6%)	14 (9.7%)	19 (13.2%)	0.46*
	Primary school	8 (5.6%)	8 (5.6%)	9 (6.3%)	
	Secondary school	11 (7.6%)	11 (7.6%)	13 (9.0%)	
	graduation	15 (10.4%)	12 (8.3%)	5 (3.5%)	
	More than graduation	3 (2.1%)	3 (2.1%)	2 (1.4%)	
**Occupation**	Professional	2 (1.4%)	3 (2.1%)	1 (0.7%)	0.44*
	Employment	15 (10.4%)	16 (11.1%)	10 (6.9%)	
	Business	8 (5.6%)	13 (9.0%)	17 (11.8%)	
	Vocational	6 (4.2%)	3 (2.1%)	3 (2.1%)	
	None	17 (11.8%)	13 (9.0%)	17 (11.8%)	
**Frequency of teeth cleaning**	Once a day	16 (11.1%)	16 (11.1%)	16 (11.1%)	0.39*
	Twice a day	15 (10.4%)	12 (8.3%)	5 (3.5%)	
	Once a week	9 (6.3%)	11 (7.6%)	13 (9.0%)	
	2–3 times a week	6 (4.2%)	6 (4.2%)	8 (5.6%)	
	Rarely	2 (4.2%)	3 (2.1%)	6 (4.2%)	
**Tool used for teeth cleaning**	Toothbrush	39 (27.1%)	36 (25.0%)	26 (18.1%)	0.05*
	Miswak	5 (3.5%)	6 (4.2%)	11 (7.6%)	
	Others	4 (2.8%)	6 (4.2%)	11 (7.6%)	
**Last dental visit**	≤ 1 year	20 (13.9%)	12 (8.3%)	20 (13.9%)	0.02*
	≥ 1 year	27 (18.8%)	29 (20.1%)	35 (24.3%)	
	None	1 (0.7%)	7 (4.9%)	5 (3.5%)	
**Reason for a dental visit**	Pain	27 (18.8%)	29 (20.1%)	34 (23.6%)	0.04*
	Sensitivity	8 (5.6%)	4 (2.8%)	3 (2.1%)	
	Scaling and polishing	11 (7.6%)	8 (5.6%)	3 (2.1%)	
	Others	1 (0.7%)	0 (0.0%)	3 (2.1%)	
	None	1 (0.7%)	7 (4.9%)	5 (3.5%)	
**Tobacco consumption and types used**	Smoked tobacco	7 (4.9%)	9 (6.3%)	6 (4.2%)	0.75
	Smokeless tobacco	8 (5.6%)	11 (7.6%)	12 (8.3%)	
	None	33 (22.9%)	28 (19.4%)	30 (20.8%)	
**Frequency of tobacco consumption**	1–2 times a day	3 (2.1%)	3 (2.1%)	3 (2.1%)	
	≥ 3 times a day	12 (83%)	17 (11.8%)	15 (10.4%)	0.86*
	None	33 (22.9%)	28 (19.4%)	30 (20.8%)	
**Duration of diabetes**	≤ 5 years	0 (0.0%)	24 (16.7%)	11 (7.6%)	0.001*
	6–9 years	0 (0.0%)	16 (11.2%)	15 (10.5%)	
	≥ 10 years	0 (3.3%)	8 (5.6%)	22 (15.4%)	
**Diabetes medication**	None	48 (33.3%)	13 (9.0%)	1 (0.7%)	0.001*
	Oral	0 (0.0%)	34 (23.6%)	34 (23.6%)	
	Combination (oral + inj insulin)	0 (0.0%)	1 (0.7%)	13 (9.0%)	
**Community Periodontal Index (CPI)**	Healthy-0	30 (20.8%)	15 (10.4%)	6 (4.2%)	0.001*
	Bleeding on probing − 1	4 (2.8%)	5 (3.5%)	4 (2.8%)	
	Calculus detected- 2	3 (2.1%)	6 (4.2%)	8 (5.6%)	
	Pocket 4–5 mm (gingival margin within the black band of probe) − 3	8 (5.6%)	12 (8.3%)	11 (7.6%)	
	Pocket ≥ 6 mm (black band not visible)-4	3 (2.1%)	10 (6.9%)	19 (13.2%)	
**Loss of Attachment Index**	Loss of attachment 0-3 mm (CEJ not visible) Code-0	27 (18.8%)	17 (11.8%)	5 (3.5%)	0.001*
	Loss of attachment 4-5 mm (CEJ within the black band) Code − 1	17 (11.8%)	15 (10.4%)	11 (7.6%)	
	Loss of attachment 6-8 mm (CEJ between 5-8 mm ring) Code-2	2 (1.4%)	7 (4.9%)	15 (10.4%)	
	Loss of attachment 9-11 mm (CEJ between 8.5-11.5 mm ring) Code- 3	1 (0.7%)	6 (4.2%)	8 (5.6%)	
	Loss of attachment ≥ 12 mm (CEJ beyond 11 mm ring)- 4	1 (0.7%)	3 (2.1%)	9 (6.3%)	
**Oral Hygiene Index- Simplified (OHI-S)**	Good oral hygiene	27 (18.8%)	19 (13.2%)	10 (6.9%)	
	Fair oral hygiene	7 (4.9%)	7 (4.9%)	9 (6.3%)	0.03
	Poor oral hygiene	14 (9.7%)	22 (15.3%)	29 (20.1%)	

Chi-square test/Fisher Exact* | p ≤ 0.05 = statistically significant | p = 0.001 = highly significant

**Table 2:** Comparative analysis of continuous variables with non-diabetic/diabetic groups

Variable	Groups	Mean	Std.Deviation	95% Confidence Interval for Mean		F	P Value
				Lower	Upper		
Age	Non-diabetic	42.54	12.33	38.95	46.12	5.46	0.005
	Controlled diabetic	43.72	10.23	40.75	46.69		
	Uncontrolled diabetic	49.22	8.87	46.65	51.8		
HbA1c level(%)	Non-diabetic	5.24	0.41	5.12	5.36	207.26	0.001
	Controlled diabetic	6.72	0.5	6.57	5.3		
	Uncontrolled diabetic	8.3	1.09	7.98	8.61		
Number of missing teeth (except 3rd molar)	Non-diabetic	1.35	1.63	0.88	1.82	6.55	0.002
	Controlled diabetic	1.7	1.79	1.18	2.23		
	Uncontrolled diabetic	2.64	1.97	2.07	3.21		

One Way ANOVA| p ≤ 0.05 = statistically significant

**Table 3:** Post Hoc (Tukey HSD) Test with Multiple comparisons

Dependent Variable	Type of groups	Intra groups Comparison	Mean Difference	P value	95% Confidence Interval	
					Lower Bound	Upper Bound
Age	Controlled T2DM	Uncontrolled T2DM	-5.50	0.03*	-10.61	-0.39
		Non-diabetic	1.18	0.85	-3.93	6.30
	Uncontrolled T2DM	Controlled T2DM	5.500	0.03*	0.39	10.61
		Non-diabetic	6.68	0.01*	1.57	11.80
	Non-diabetic	Controlled T2DM	-1.18	0.85	-6.30	3.93
		Uncontrolled T2DM	-6.68	0.01*	-11.80	-1.57
Level of HbA1c (%)	Controlled Diabetes	Uncontrolled T2DM	-1.57	0.00*	-1.93	-1.22
		Non-diabetic	1.47	0.00*	1.12	1.83
	Uncontrolled T2DM	Controlled T2DM	1.57	0.00*	1.22	1.93
		Non-diabetic	3.05	0.00*	2.70	3.41
	Non-diabetic	Controlled T2DM	-1.47	0.00*	-1.83	-1.12
		Uncontrolled T2DM	-3.05	0.00*	-3.41	-2.70
Number of missing teeth (except 3rd molar)	Controlled T2DM	Uncontrolled T2DM	− 0.93	0.03*	-1.81	-0.06
		Non-diabetic	0.35	0.60	-0.52	1.23
	Uncontrolled T2DM	Controlled T2DM	0.93	0.03*	0.06	1.81
		Non-diabetic	1.29	0.00*	0.42	2.16
	Non-diabetic	Controlled T2DM	− 0.35	0.60	-1.23	0.52
		Uncontrolled T2DM	-1.29	0.00*	-2.16	-0.42

*. The mean difference is significant at the 0.05 level

### Periodontal Status among Non-Diabetics, controlled diabetics, and uncontrolled diabetics

**Fig. 1:**
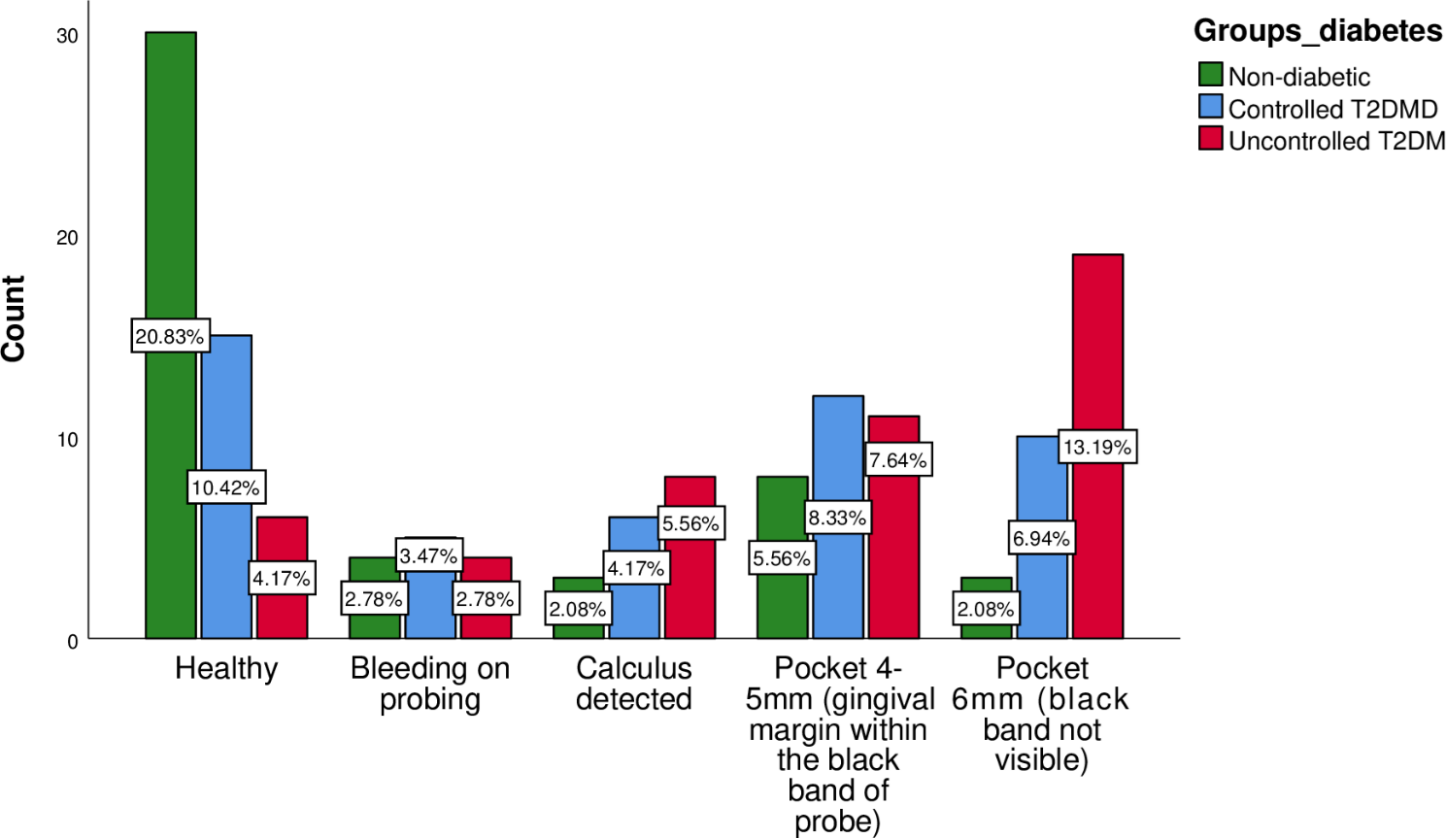
Community Periodontal Index

**Fig. 2:**
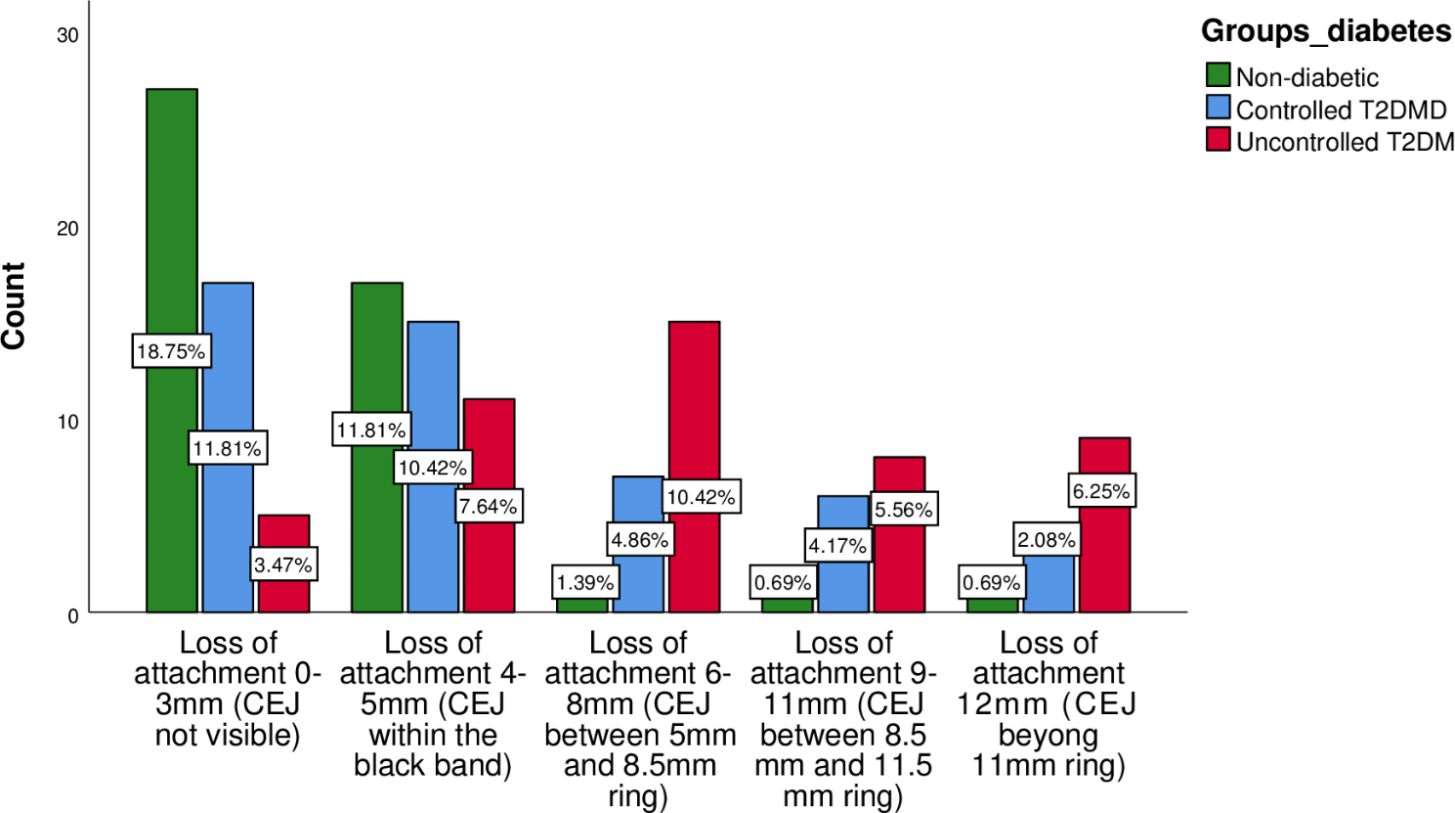
Loss of Attachment Index

Periodontal status as clinically determined via the Community Periodontal Index, Loss of Attachment Index, and missing teeth, varied distinctly among the three groups. Healthy periodontium (code-0) was found in 30 (20.8%) of non-diabetics while this ratio was 6 (4.2%) in uncontrolled T2DM (p = 0.001). Similarly, code-4 was most prevalent in uncontrolled T2DM 19 (13.2%); p = 0.01 and least in the non-diabetic group 1(0.7%) (Table 1 and Fig. 1). Loss of attachment (code-0) was prevalent in non-diabetics [27 (18.8%); p = 0.001] followed by the controlled T2DM group [17 (11.8%); p = 0.001] and infrequent in uncontrolled T2DM [5 (3.5%); p = 0.001]. Loss of attachment (code-4) was more common in the uncontrolled T2DM group [9 (6.3%); p = 0.001] and least common in non-diabetics [1 (0.7%); p = 0.001] (Table 1 and Fig. 2). The uncontrolled T2DM group showed the highest mean for missing teeth [2.64 ± 1.97; p = 0.01] followed by controlled T2DM [1.70 ± 1.79; p = 0.01] and non-diabetics [1.35 ± 1.63; p = 0.01] (Table 1).

### Oral hygiene status among non-diabetics, controlled diabetics, and uncontrolled diabetics

**Fig. 3:**
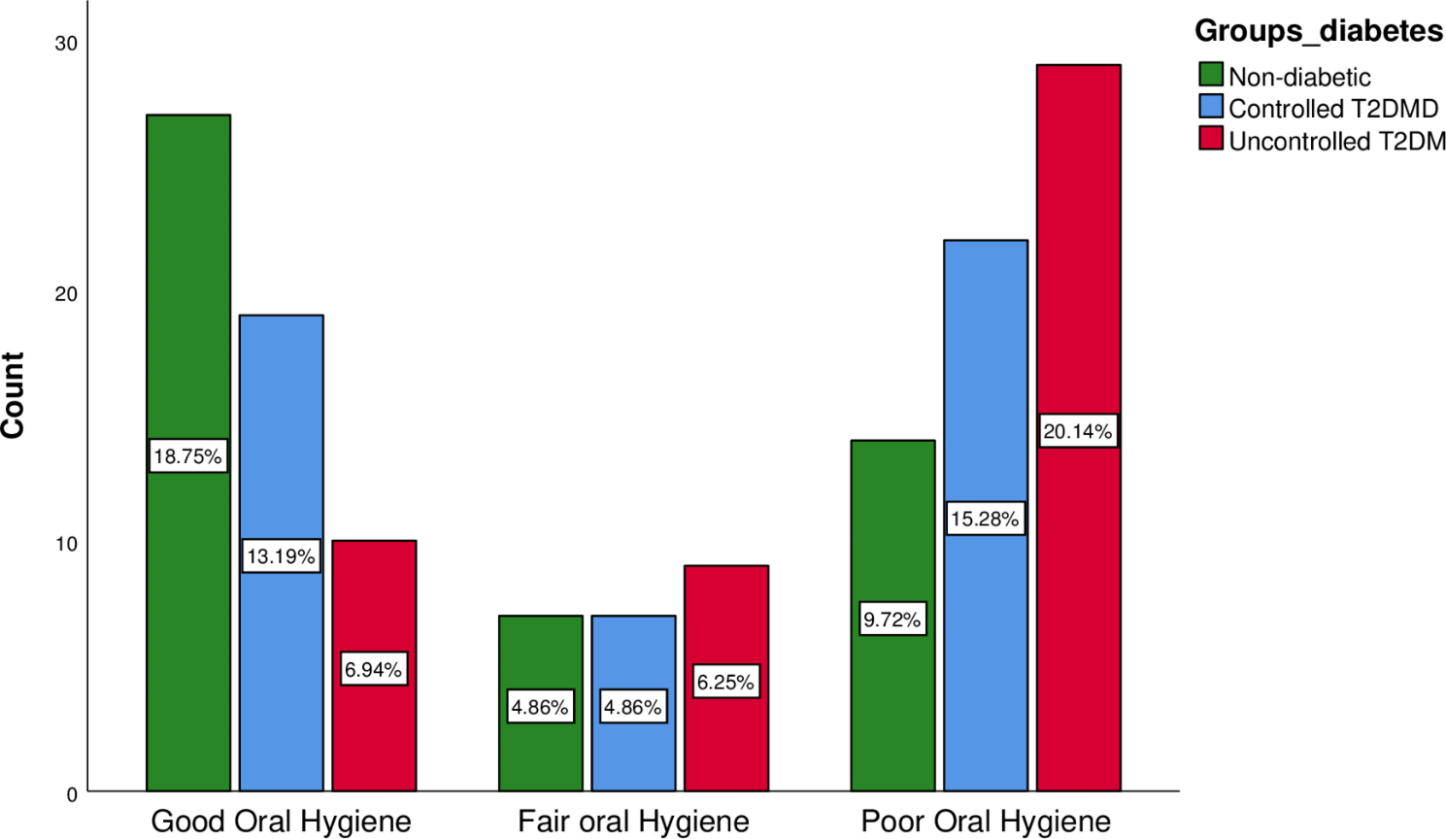
Oral Hygiene Index -Simplified

Oral hygiene status was designated as Good, Fair, and Poor according to Oral Hygiene Index- Simplified. Good Oral Hygiene was observed in [27 (18.8%); p = 0.03] non-diabetics followed by controlled T2DM [19 (1.2%); p = 0.03] and uncontrolled T2DM [09 (6.3%); p = 0.03]. Poor oral hygiene was more prevalent in uncontrolled T2DM [29 (20.1%; p = 0.03) and least prevalent in non-diabetics [14 (9.7%); p = 0.03] (Tables 1 and Fig. 3).

## Discussion

By utilizing a representative sample of adults with type II diabetes mellitus and those without the disease, a statistically significant association was found between periodontal and oral hygiene status and the HbA1c levels. The results of this study showed that patients with uncontrolled T2DM had worse periodontal health as revealed by the increase in bleeding on probing and pocket depth (assessed by CPI Index) and increased clinical attachment loss (assessed by Loss of Attachment Index) as compared to their healthy counterpart and controlled T2DM.

CPI code 0 (healthy) was more prevalent in non-diabetics and controlled T2DM as compared to uncontrolled T2DM which are close to the observations of the other studies comparing the same groups [18–20]. Similarly, code 1 (BOP) was almost similar among the three groups, which opposes the findings of other cross-sectional studies, using the CPI index [21–23] and coincides with the study of Kim et al. [19]. CPI code 2 (calculus) was higher in uncontrolled T2DM in comparison to non-diabetics and controlled T2DM which is consistent with the results of *Apporva et al.* [20]. The code 2 scores of our study contradict the results of other cross-sectional studies involving T2DM patients [19, 24]. The three groups of the present study shared almost the same scores for CPI code 3, which contradict the results of studies done by other investigators involving CPI usage [18, 20]. CPI code 4 was highest in uncontrolled T2DM as compared to non-diabetics and controlled T2DM, which is alike the findings of other studies [18–20, 22]. The tentative propositions for these similarities and discrepancies could be the same/different sociodemographic characteristics, oral hygiene behavior, education level, access to dental services, and discrepancies/similarities in the measuring. On a universally agreeable scale, the possible phenomenon resulting in these grievous periodontal clinical parameters is the diabetes-periodontitis reciprocality. T2DM impacts periodontitis development, progression, and severity by the production of advanced glycation end products, triggering a hyperinflammatory response, modulation of the periodontal microbiota, and impaired alveolar bone healing [25–28].

Clinical attachment loss, which is another prime indicator of periodontal disease progression, was also assessed among the three groups in this study. Loss of attachment code 0 (Loss of attachment 0-3 mm) was highest in non-diabetics (18.8%) compared to uncontrolled T2DM patients (3.5%), while code 4 (Loss of attachment 9-11 mm) which is the worst score, was highest in uncontrolled T2DM (6.3%) compared to non-diabetics in which it was prevalent in just 0.7%. These findings are supported by other studies which compared the same among T2DM patients [28, 29]. The possible mechanisms which affect the clinical level are AGEs which initiate and progress a cascade of inflammatory events resulting in the degradation of the attachment apparatus. Moreover, the fibroblasts’ function is impaired by the hyperglycemic environment, predisposing the collagen to degrade, which inhabits the repair and regeneration [29, 30].

Another key finding of this study was the increased number of tooth loss in uncontrolled T2DM. This finding is in accordance with previous studies which proved that tooth loss due to periodontitis is closely influenced by uncontrolled T2DM. This is supported by other studies [31, 32] The possible explanation for this augmented tooth loss in uncontrolled T2DM is the defective bone composition and structure as well as increased severity of alveolar bone loss [33, 34]. Tooth loss due to periodontitis not only jeopardizes the masticatory function but also negatively affects the levels of progenitor cells which appear to increase the susceptibility of endothelial cell dysfunction and chronic inflammation [35].

Oral hygiene was another prime aspect of this cross-sectional study. Oral hygiene was determined with the Oral Hygiene Index Simplified which is a simple and time-saving tool with three categories of Good, Fair, and Poor Oral hygiene. Non-diabetics had good oral hygiene as compared to uncontrolled T2DM, while poor oral hygiene was more prevalent in uncontrolled T2DM in contrast to non-diabetics. Other studies comparing oral hygiene status are consistent with the finding of these studies [6, 31]. These findings contradict the results of a cross-sectional study done in India, in which only 22% of diabetics showed poor oral hygiene while 37% of non-diabetics showed poor oral hygiene [24]. The possible justification for this contrast could be differences in oral hygiene behavior like less frequency of tooth brushing, improper tooth brushing technique or lack of proper oral hygiene knowledge, or compromised access to dental care services. Poor oral hygiene and low frequency of toothbrushing not only enhance the prevalence of periodontal disease but also increase the risk of T2DM [36, 37].

This study attempted to comprehend the two key risk factors (periodontitis and T2DM) of oral and systemic health. Both these conditions, sharing a bidirectional relationship, require a multidisciplinary approach to minimize and prevent the adverse effects of these two chronic diseases on oral health and overall well-being. Dentists should be aware of the diabetic status of their patients and the same goes for the physicians/diabetologist to be aware of the oral complication of diabetes mellitus. Access to dental care should be made easier and regular professional dental care should be encouraged in T2DM patients specifically, which will not only minimize the diabetes’ oral complication but also will improve the serum HbA1c levels [27, 38–40].

The limitation of this study was the sample size which was limited to a specific geographic area that cannot be generalized. A cross-sectional study could be the other limitation. Using the WHO-validated tool and oral examination by a single examiner were the strengths of this study. Future studies may be carried out on a large sample size using different study designs to find out a temporal relationship between periodontal health and T2DM.

## Conclusion

In light of the results of this cross-sectional study, it is concluded that periodontal parameters (bleeding on probing, pocket depth, clinical attachment loss, and tooth loss) and oral hygiene status (plaque and calculus) were worsened in uncontrolled in T2DM in comparison to non-diabetic participants. A multidisciplinary approach is needed to maintain periodontal health and overall well-being in diabetic patients.

## Electronic supplementary material

Below is the link to the electronic supplementary material.

Supplementary Material 1

Supplementary Material 2

## Data Availability

All data generated or analyzed during this study are included in these published articles & supporting materials (Supporting data_S1 and Supporting data_S2).

## List of Abbreviations

HbA1c: Hemoglobin A1c
T2DM: Type 2 diabetes mellitus
WHO: World Health Organization
CPITN-C: Community Periodontal Index of Treatment Needs - C
CPI: Community Periodontal Index
OHI- S: Oral Hygiene Index – Simplified
BOP: Bleeding of probing
AGEs: Advanced Glycation End Products

## References

[1] Slots J. Periodontitis: facts, fallacies and the future. Periodontol 2000. 2017;75(1):7–23. 10.1111/prd.12221 28758294

[2] Thornton-Evans G, Eke P, Wei L, Palmer A, Moeti R, Hutchins S, et al. Periodontitis among adults aged ≥ 30 years - United States, 2009–2010. MMWR Suppl. 2013;22(3):129–35. 24264502

[3] The Institute for Health Metrics and Evaluation (IHME). Periodontal diseases — Level 4 cause [Internet]. [cited 2023 Apr 4]. Available from: https://www.healthdata.org/results/gbd_summaries/2019/periodontal-diseases-level-4-cause.

[4] Hajishengallis G, Chavakis T. Local and systemic mechanisms linking periodontal disease and inflammatory comorbidities. Nat Rev Immunol 2021;21(7):426–40. 10.1038/s41577-020-00488-6 PMC7841384 33510490

[5] Isola G, Santonocito S, Distefano A, Polizzi A, Vaccaro M, Raciti G et al. Impact of periodontitis on gingival crevicular fluid miRNAs profiles associated with cardiovascular disease risk. J Periodontal Res 2023 Feb 8;58(1):165–74. 10.1111/jre.13078 36482859

[6] Isola G, Giudice A, Lo, Polizzi A, Alibrandi A, Patini R, Ferlito S. Periodontitis and Tooth Loss Have Negative Systemic Impact on Circulating Progenitor Cell Levels: A Clinical Study. Genes (Basel). 2019;10(12):01–12. 10.3390/genes10121022 PMC6947645 31817862

[7] Ronnie Levine, Catherine Stillman-Lowe. The scientific basis of oral Health Education (BDJ Clinician’s Guides). 8th ed. Springer; 2018.

[8] Petersen PE, Baez RJ, Organization WH. Oral health surveys: basic methods [Internet]. 5th ed. Geneva: World Health Organization; 2013. Available from: https://apps.who.int/iris/handle/10665/97035.

[9] Lertpimonchai A, Rattanasiri S, Arj-Ong Vallibhakara S, Attia J, Thakkinstian A. The association between oral hygiene and periodontitis: a systematic review and meta-analysis. Int Dent J [Internet]. 2017 Dec 1 [cited 2023 Apr 9];67(6):332–43. Available from: https://pubmed.ncbi.nlm.nih.gov/28646499/. 10.1111/idj.12317 PMC5724709 28646499

[10] Sun H, Saeedi P, Karuranga S, Pinkepank M, Ogurtsova K, Duncan BB et al. IDF Diabetes Atlas: Global, regional and country-level diabetes prevalence estimates for 2021 and projections for 2045. Diabetes Res Clin Pract [Internet]. 2022;183:109119. Available from: https://www.sciencedirect.com/science/article/pii/S0168822721004782. 10.1016/j.diabres.2021.109119 PMC11057359 34879977

[11] Khan MAB, Hashim MJ, King JK, Govender RD, Mustafa H, Al Kaabi J (2019). Epidemiology of type 2 diabetes – global burden of Disease and Forecasted Trends. J Epidemiol Glob Health.

[12] Khader YS, Dauod AS, El-Qaderi SS, Alkafajei A, Batayha WQ. Periodontal status of diabetics compared with nondiabetics: a meta-analysis. J Diabetes Complications. 2006;20(1):59–68. 10.1016/j.jdiacomp.2005.05.006 16389170

[13] Genco RJ, Grossi SG, Ho A, Nishimura F, Murayama Y. A proposed model linking inflammation to obesity, diabetes, and Periodontal Infections. J Periodontol. 2005;76(11–s):2075–84. 10.1902/jop.2005.76.11-S.2075 16277579

[14] Preshaw PM, Taylor JJ, Jaedicke KM, De Jager M, Bikker JW, Selten W et al. Treatment of periodontitis reduces systemic inflammation in type 2 diabetes. J Clin Periodontol. 2020;47(6):737–46. 10.1111/jcpe.13274 32106333

[15] Pattayil S, Vadakkekuttical RJ, Radhakrishnan C, Kanakkath H, Hrishi TS. Proportional relationship between periodontal inflamed surface area, clinical attachment loss, and glycated hemoglobin level in patients with type 2 diabetes mellitus on insulin therapy and on oral antidiabetic therapy. J Periodontol. 2023;3(1):31–40. 10.1002/JPER.22-0085 35716397

[16] Banjar A, Alyafi R, AlGhamdi A, Assaggaf M, Almarghlani A, Hassan S et al. The relationship between glycated hemoglobin level and the stage of periodontitis in individuals without diabetes. PLoS One 2023;18(1):e0279755. 10.1371/journal.pone.0279755 PMC9821507 36608039

[17] Zhang X, Wang M, Wang X, Qu H, Zhang R, Gu J et al. Relationship between periodontitis and microangiopathy in type 2 diabetes mellitus: a meta-analysis. J Periodontal Res 2021;56(6):1019–27. 10.1111/jre.12916 34254680

[18] Kesavan R, Chaly P, Reddy Vc M, Av. Periodontal status among type II diabetic and nondiabetic individuals in Chennai, India: a comparative study. J Indian Association Public Health Dentistry. 2015;13(4):393–8.

[19] SingleCare Team. The 411 on A1C: Normal A1C levels and 15 ways to lower high A1C [Internet]. 2020 [cited 2023 Apr 16]. Available from: https://www.singlecare.com/blog/normal-a1c-levels/.

[20] Reddy CVK, Maurya M. A comparative study to assess the oral health status and treatment needs of diabetics and non-diabetic population attending some of the hospitals in Mysore City. Journal of Indian Association of Public Health Dentistry [Internet]. 2023 [cited 2023 Apr 14];6(12):1–14. Available from: https://journals.lww.com/aphd/pages/default.aspx/article.asp?issn=2319-5932;year=2008;volume=6;issue=12;spage=1;epage=14;aulast=Reddy;type=0.

[21] Kim EK, Lee SG, Choi YH, Won KC, Moon JS, Merchant AT et al. Association between diabetes-related factors and clinical periodontal parameters in type-2 diabetes mellitus. BMC Oral Health 2013;13(1):1–8. 10.1186/1472-6831-13-64 PMC3829373 24195646

[22] Apoorva S, Sridhar N, Suchetha A. Prevalence and severity of periodontal disease in type 2 diabetes mellitus (non-insulin-dependent diabetes mellitus) patients in Bangalore city: an epidemiological study. J Indian Soc Periodontol. 2013;17(1):25–9. 10.4103/0972-124X.107470 PMC3636938 23633768

[23] Khader YS, Albashaireh ZSM, Hammad MM (2008). Periodontal status of type 2 diabetics compared with nondiabetics in north Jordan. East Mediterr Health J.

[24] Mohamed HG, Idris SB, Ahmed MF, Bøe OE, Mustafa K, Ibrahim SO et al. Association between Oral Health Status and Type 2 Diabetes Mellitus among Sudanese Adults: A Matched Case-Control Study. PLoS One [Internet]. 2013 Dec 11 [cited 2023 Apr 14];8(12):e82158. Available from: https://journals.plos.org/plosone/article?id=10.1371/journal.pone.0082158. 10.1371/journal.pone.0082158 PMC3859584 24349205

[25] Javed F, Näsström K, Benchimol D, Altamash M, Klinge B, Engström PE. Comparison of Periodontal and Socioeconomic Status between subjects with type 2 diabetes Mellitus and non-diabetic controls. J Periodontol. 2007;78(11):2112–9. 10.1902/jop.2007.070186 17970677

[26] Chee B, Park B, Bartold MP. Periodontitis and type II diabetes: a two-way relationship. Int J Evid Based Healthc. 2013;11(4):317–29. 10.1111/1744-1609.12038 24298927

[27] Lalla E, Papapanou PN. Diabetes mellitus and periodontitis: a tale of two common interrelated diseases. Nat Rev Endocrinol 2011;7(12):738–48. 10.1038/nrendo.2011.106 21709707

[28] Teles F, Wang Y, Hajishengallis G, Hasturk H, Marchesan JT. Impact of systemic factors in shaping the periodontal microbiome. Periodontol 2000. 2021;85(1):126–60. 10.1111/prd.12356 33226693

[29] Wu C, Yuan Y hang, Liu H hang, Li S sui, Zhang B wen, Chen W et al. Epidemiologic relationship between periodontitis and type 2 diabetes mellitus. BMC Oral Health. 2020;20(1):01–15. 10.1186/s12903-020-01180-w PMC7353775 32652980

[30] Preshaw PM, Alba AL, Herrera D, Jepsen S, Konstantinidis A, Makrilakis K et al. Periodontitis and diabetes: a two-way relationship. Diabetologia 2012;55(1):21–31. 10.1007/s00125-011-2342-y PMC3228943 22057194

[31] Buranasin P, Mizutani K, Iwasaki K, Pawaputanon Na Mahasarakham C, Kido D, Takeda K et al. High glucose-induced oxidative stress impairs proliferation and migration of human gingival fibroblasts. PLoS One. 2018;13(8):e0201855. 10.1371/journal.pone.0201855 PMC6084939 30092096

[32] Sandini Trentin M, De João; P, De M, Ferreira C, Diego ;, et al. Prevalence and Severity of Periodontal Disease in Type 2 Diabetes Mellitus Patients: a Cross-Sectional Study. Biosci J. 2018;4(34):1114–23.

[33] Kaur G, Holtfreter B, Rathmann WG, Schwahn C, Wallaschofski H, Schipf S et al. Association between type 1 and type 2 diabetes with periodontal disease and tooth loss. J Clin Periodontol [Internet]. 2009 Sep 1;36(9):765–74. Available from: 10.1111/j.1600-051X.2009.01445.x. 10.1111/j.1600-051X.2009.01445.x 19622096

[34] Pabisch S, Akabane C, Wagermaier W, Roschger A, Ogura T, Hyodo R, et al. The nanostructure of murine alveolar bone and its changes due to type 2 diabetes. J Struct Biol. 2016;196(2):223–31. 10.1016/j.jsb.2016.09.007 27637572

[35] Farooq A, Riasat M, Baloch HKN, Shah SGS, Shehzad S, Shah SZEHS. Severity of alveolar bone loss in control and uncontrolled type II diabetics. Pakistan J Med Health Sci 2021;15(6):1459–62.

[36] Hintao J, Teanpaisan R, Chongsuvivatwong V, Dahlen G, Rattarasarn C. Root surface and coronal caries in adults with type 2 diabetes mellitus. Community Dent Oral Epidemiol. 2007;35(4):302–9. 10.1111/j.1600-0528.2007.00325.x 17615017

[37] Chang Y, Lee JS, Lee KJ, Woo HG, Song TJ. Improved oral hygiene is associated with decreased risk of new-onset diabetes: a nationwide population-based cohort study. Diabetologia. 2020;63(2):924–33. 10.1007/s00125-020-05112-9 32128623

[38] Fu W, Lv C, Zou L, Song F, Zeng X, Wang C et al. Meta-analysis on the association between the frequency of tooth brushing and diabetes mellitus risk. Diabetes Metab Res Rev 2019;35(5):e3141. 10.1002/dmrr.3141 30758127

[39] Nishihara U, Tanabe N, Nakamura T, Okada Y, Nishida T, Akihara S. A periodontal disease care program for patients with type 2 diabetes: a randomized controlled trial. J Gen Fam Med. 2017;18(5):249–57. 10.1002/jgf2.58 PMC5689414 29264035

[40] Greenblatt AP, Salazar CR, Northridge ME, Kaplan RC, Taylor GW, Finlayson TL et al. Association of diabetes with tooth loss in Hispanic/Latino adults: findings from the Hispanic Community Health Study/Study of Latinos. BMJ Open Diabetes Res Care. 2016;4(1):e000211. 10.1136/bmjdrc-2016-000211 PMC4873949 27239319

