# Supplementary material for: Association and comparison of periodontal and oral hygiene status with serum HbA1c levels: a cross-sectional study
Source: BMC Oral Health. 2023 Jul 2;23:442. doi: 10.1186/s12903-023-03042-7 (PMC10316548; doi:10.1186/s12903-023-03042-7)
Supplement: Supplementary file 1 — Supplementary Material 1 [file 12903_2023_3042_MOESM1_ESM.pdf]

**COMPARISON OF PERIODONTAL AND ORAL HYGIENE STATUS OF NON-DIABETICS AND TYPE-II DIABETICS:**
**A CROSS-SECTIONAL STUDY IN KHYBER PAKHTUNKHWA, PAKISTAN**

***Supporting data_S1***

**Annexure I: Demographic, Oral Health/Diabetes related questionnaire (modified from WHO oral health survey)**

Date: / /

1. Name:
2. Gender:
3. OPD/Reg no:
4. Age:
5. Education:
6. Occupation
7. Income: ___________________ PKR/month
8. Diabetic/non-diabetic:
9. How often do you clean your teeth?
10. What do you use to clean your teeth?
11. How long is it since you last visit a dentist?
12. What was the reason of your last visit to the dentist?
13. What tobacco product do you use?
14. How often do you use that tobacco product?
15. Duration of diabetes

    ≤5 years
    6-9 years
    ≥10 years

16. HbA1c % (A/B/C)

    A. ≤5.7

    B. 6 – 6.9

    C. ≥7

17. Diabetes (T2D) medication.

    A. Oral only B. Insulin inj.

    C. Combination

18. T2D Complications

    A. Yes B. No

19. Any other medication use/d?

    A. Yes B. No

20. Any other treatment undergone?

    A. Yes B. No

**RECORDING CHARTS (WHO):**

## 1. COMMUNITY PERIODONTAL INDEX (CPI)

Adapted from WHO/FDI in 1982

a. Community Periodontal Index (CPI):

| 17/16 | 11 | 26/27 |
|---|---|---|
| | | |
| | | |
| 47/46 | 31 | 36/37 |

| Score | Criteria |
|---|---|
| 0 | Healthy |
| 1 | Bleeding observed |
| 2 | Calculus detected |
| 3 | Pocket 4-5 mm (gingival margin within the black band of the probe) |
| 4 | Pocket ≥ 6mm (black band not visible) |
| X | Excluded sextant (< 2 teeth present) |
| 9 | Tooth not present |

b. Loss of Attachment (Clinical attachment loss):

| 17/16 | 11 | 26/27 |
|---|---|---|
| | | |
| | | |
| 47/46 | 31 | 36/37 |

| Score | Criteria |
|---|---|
| 0 | Loss of attachment 0-3mm (CEJ not visible) |
| 1 | Loss of attachment 4-5mm (CEJ within the black band) |
| 2 | Loss of attachment 6-8mm (CEJ between 5. mm and 8.5mm ring) |
| 3 | Loss of attachment 9-11mm (CEJ between 8.5mm and 11.5mm ring) |
| 4 | Loss of attachment ≥12mm (CEJ beyond 11mm ring) |
| X | Excluded sextant (<2 teeth present) |
| 9 | Not present |

Number of missing teeth (except 3rd molar):

________

## 2. ORAL HYGIENE INDEX-SIMPLIFIED (OHI-S):

Described by John C. Greene and Jack R. Vermillion in 1964
Comprises of Debris Index–Simplified (DI-S) and Calculus Index–Simplified (CI-S)

| Tooth | DI-S | CI-S |
|---|---|---|
| | | |
| 16 | | |
| 11 | | |
| 26 | | |
| 36 | | |
| 31 | | |
| 46 | | |
| Total | | |
| Score | | |
| OHI-S | | |

OHI-S = DI-S + CI-S

Interpretation:

| Score | Criteria |
|---|---|
| 0 | No debris/plaque or stains present |
| 1 | Plaque covering ≤1/3 of tooth surface or presence of stains regardless of plaque presence |
| 2 | Plaque covering >1/3 and <2/3 of the tooth surface |
| 3 | Plaque covering > 2/3 of the tooth surface |

| Score | Criteria |
|---|---|
| 0 | No calculus presents |
| 1 | Supragingival calculus covering ≤1/3 of tooth surface |
| 2 | Supragingival calculus covering >1/3 and <2/3 of the tooth surface and/or presence of individual flecks of subgingival calculus around the cervical portion of the tooth |
| 3 | Supragingival calculus covering > 2/3 of the tooth surface and/or continuous heavy band of subgingival calculus around the cervical portion of the tooth |

*Interpretation*

**An OHI-S is scored as follows:**

0.0–1.2 = Good oral hygiene
1.3–3.0 = Fair oral hygiene
3.1–6.0 = Poor oral hygiene
